# Regulation of GLI2 by LINC02560 and its role in hepatocellular carcinoma

**DOI:** 10.1007/s12672-025-03162-2

**Published:** 2025-07-18

**Authors:** Yunmei Huang, Zhang Qingyun, Chunying Luo

**Affiliations:** 1 Department of Pathology, Affiliated Hospital of Youjiang Medical University for Nationalities, Baise, China; 2 Key Laboratory of Molecular Pathology in Tumors of Guangxi Higher Education Institutions, Baise, China; 3 Youjiang Medical University for Nationalities, Baise, China

**Keywords:** LINC02560, GLI2, HCC, LncRNA

## Abstract

This article focuses on the regulation of GLI2 by LINC02560 in HCC. Revealing this mechanism provides a new perspective for the morbidity mechanism and treatment strategy of hepatocellular carcinoma.LINC02560, a long non-coding RNA (lncRNA), is upregulated in hepatocellular carcinoma through interaction with GLI2 protein. They jointly regulate key biological processes such as cell proliferation, invasion, migration and apoptosis. It was found that LINC02560 not only directly binds to GLI2 protein, It also plays a regulatory role by affecting the epigenetic modification of GLI2 gene and the interaction with other transcription factors. These mechanisms contribute to the initiation and progression of HCC. In addition, the expression level of LINC02560 is closely related to the therapeutic effect and prognosis of hepatocellular carcinoma. It provides an important basis for the formulation of individualized treatment plan. Therefore, the study of GLI2 regulation by LINC02560 not only deepens our understanding of the mechanism of morbidity in hepatocellular carcinoma, It also provides important clues for finding new therapeutic targets and improving therapeutic effects.

## Introduction

Hepatocellular Carcinoma (HCC) is one of the most common malignant tumors in the world. Its morbidity and mortality are high, which poses a serious threat to human health. Although significant progress has been made in the diagnosis and treatment of hepatocellular carcinoma in recent years, However, there are still many unsolved mysteries about its morbidity mechanism and therapeutic effect. Therefore, it is necessary to further explore the morbidity mechanism of hepatocellular carcinoma and find new therapeutic targets [1]. It is of great significance to improve the survival rate and quality of life of patients with hepatocellular carcinoma.

Abnormal regulation of transcription factors has always been a hot topic in the study of hepatocellular carcinoma. As an important component of the Hedgehog signaling pathway, GLI2 transcription factor plays a crucial role in embryonic development, tissue repair, and tumorigenesis [2]. In recent years, an increasing number of studies have shown that abnormal activation of GLI2 is closely related to the occurrence and development of hepatocellular carcinoma [3]. However, the specific regulatory mechanism of GLI2 in hepatocellular carcinoma has not been fully elucidated, and its upstream regulatory factors also urgently need to be further studied.

As a long non-coding RNA (lncRNA), LINC02560 shows abnormal expression patterns in a variety of cancers. And participate in the occurrence and development of cancer. Although there are relatively few studies on LINC02560 in hepatocellular carcinoma, it has been shown that. The abnormal expression of LINC02560 is closely related to the malignant biological behavior of hepatocellular carcinoma [4]. More importantly, recent studies suggest that LINC02560 plays an important role in hepatocellular carcinoma by regulating GLI2.This discovery not only provides a new perspective for us to further understand the morbidity mechanism of hepatocellular carcinoma, It also provides strong support for finding new therapeutic targets [5].

The aim of this study was to investigate the expression and function of LINC02560 in hepatocellular carcinoma, and to elucidate the mechanism of its regulation of GLI2.Through this study, we expect to provide new ideas and methods for the treatment of hepatocellular carcinoma. It brings better therapeutic effect and survival prognosis for patients. At the same time, this study will also provide a new perspective and inspiration for the study of long non-coding RNA in cancer, and promote the development of related fields.

## Expression and function of LINC02560 in hepatocellular carcinoma

### Basic features of LINC02560

LINC02560, as a lncRNA, has gradually emerged in cancer research in recent years. Such RNA molecules are usually more than 200 nucleotides in length and do not encode proteins. However, it can regulate gene expression through various mechanisms at the transcriptional and post-transcriptional levels [6]. As a member of lncRNA family, LINC02560 has a series of unique biological characteristics and functions. These characteristics make it an important candidate molecule for the study of the morbidity mechanism of HCC [7].

LINC02560 is located in a specific region of the human genome, Its precise chromosomal localization provides us with a starting point to study its expression and regulatory mechanisms [8]. Through bioinformatics analysis, we can obtain the nucleotide sequence of LINC02560 and find that there are many regulatory elements in its sequence. Such as promoters, enhancers, and splice sites [9]. The presence of these elements implies that LINC02560 is subject to complex transcriptional regulation and thus is expressed in specific time and space [10].

LINC02560 exhibits a specific expression pattern in a variety of tissues and cell types.LINC02560 expression levels are generally significantly higher in hepatocellular carcinoma than in normal liver tissue, This indicates that it is closely related to the occurrence and development of hepatocellular carcinoma [11]. Further studies showed that the expression of LINC02560 was regulated by many factors, including transcription factors, miRNAs and epigenetic modifications. These regulatory mechanisms act together on the promoter and transcripts of LINC02560, thus affecting its expression level in hepatocellular carcinoma [12].

LINC02560, as a functional lncRNA, plays multiple roles in hepatocellular carcinoma. First, it can interact with DNA, RNA or protein to form a complex regulatory network. Thus affecting the proliferation, migration, invasion and apoptosis of hepatocellular carcinoma cells [13]. Secondly, LINC02560 can also bind to specific miRNAs as ceRNA (competitive endogenous RNA).So as to relieve the inhibitory effect of miRNA on other target genes, and then regulate the progress of hepatocellular carcinoma [14]. In addition, LINC02560 is also involved in the metabolic regulation, immune escape and drug resistance of hepatocellular carcinoma. The diversity of these functions makes it an important target for studying the mechanism of morbidity in hepatocellular carcinoma.

Although the study of LINC02560 in hepatocellular carcinoma is still in its infancy, compared with other studied lncRNAs, It still exhibits some unique features [15]. For example, LINC02560 has a more specific expression pattern and a more complex regulatory mechanism in hepatocellular carcinoma. In addition, the functions of LINC02560 are more diverse, involving not only basic biological processes such as cell proliferation and apoptosis, It also involves advanced biological functions such as metabolic regulation and immune escape [16]. These characteristics make LINC02560 an important candidate molecule for studying the morbidity mechanism of hepatocellular carcinoma.

### Expression of LINC02560 in hepatocellular carcinoma

As an emerging lncRNA, LINC02560 has attracted more and more attention in HCC research in recent years. Through a series of experiments and data analysis, Long Puze et al. Found that the expression of LINC02560 in hepatocellular carcinoma has significant characteristics. These characteristics not only reveal its important role in the mechanism of morbidity of hepatocellular carcinoma, It also provides new ideas for the diagnosis and treatment of hepatocellular carcinoma [17].

Firstly, By comparing the expression level of LINC02560 in hepatocellular carcinoma tissues with that in normal liver tissues, Meng Fangang et al. Found that the expression of LINC02560 in hepatocellular carcinoma tissues was significantly higher than that in normal liver tissues. This finding suggests that the abnormal expression of LINC02560 is closely related to the occurrence and development of hepatocellular carcinoma. Further studies have found that, The high expression of LINC02560 was positively correlated with the clinicopathological characteristics of HCC, such as the degree of malignancy, tumor size, vascular invasion and lymph node metastasis This further confirms the important role of LINC02560 in hepatocellular carcinoma [18].

To further understand the expression of LINC02560 in hepatocellular carcinoma, Chen Hualin et al. also analyzed the relationship between LINC02560 expression level and clinicopathological features of hepatocellular carcinoma [19]. A large number of samples of hepatocellular carcinoma patients were collected and the expression level of LINC02560 was detected by real-time fluorescence quantitative PCR. Li Yi and Shang Li found that the high expression of LINC02560 was closely related to the clinicopathological features of hepatocellular carcinoma, such as TNM stage, pathological grade and prognosis [20]. The high expression of LINC02560 often indicates the poor prognosis of hepatocellular carcinoma patients. This suggests that LINC02560 is an important prognostic marker for HCC.

In addition to the relationship with clinicopathological features, Chen Yang followed up the survival of patients with hepatocellular carcinoma. Combined with the expression level of LINC02560,High expression of LINC02560 was found to be strongly associated with short survival in HCC patients [21]. This finding further confirms the value of LINC02560 in prognostic assessment of hepatocellular carcinoma.LINC02560 has become an important target for the treatment of hepatocellular carcinoma.

In order to explore the molecular mechanism of differential expression of LINC02560 in hepatocellular carcinoma, Shi Gang also conducted a series of experimental studies [22]. By means of RNA sequencing, gene chip and bioinformatics analysis, Li Bofei found that the expression of LINC02560 is regulated by many factors, including transcription factors, miRNA and epigenetic modification [23]. These regulatory factors act on the promoter, transcript and translation process of LINC02560.Thereby affecting the expression level of the protein in the hepatocellular carcinoma. These findings not only provide important clues for us to deeply understand the expression mechanism of LINC02560 in hepatocellular carcinoma, It also provides new ideas and methods for the treatment of hepatocellular carcinoma.

### Functional study of LINC02560 in hepatocellular carcinoma

LINC02560 has multiple function in hepatocellular carcinoma, These functions are not only related to the basic biological behaviors such as proliferation, migration and invasion of cancer cells, It also involves advanced biological processes such as metabolic regulation, immune escape and drug resistance of cancer cells. Through further study on the function and mechanism of LINC02560 in hepatocellular carcinoma, We can provide new ideas and methods for the diagnosis and treatment of hepatocellular carcinoma. It brings better therapeutic effect and survival prognosis for patients. Functional studies in HCC have received increasing attention. Through a series of experiments and data analysis, the multiple functions of LINC02560 in hepatocellular carcinoma were gradually revealed. These functions are not only related to the basic biological behaviors such as proliferation, migration and invasion of cancer cells, It also involves advanced biological processes such as metabolic regulation, immune escape and drug resistance of cancer cells.

LINC02560 was found to have an important cancer-promoting effect in hepatocellular carcinoma. Through in vitro cell experiments and animal model experiments, They found that overexpression of LINC02560 significantly promoted the proliferation and invasion of hepatocellular carcinoma cells. This finding suggests that LINC02560 accelerates the progression of hepatocellular carcinoma by regulating the growth and migration of cancer cells [24]. Further studies have found that LINC02560 regulates transcription factors related to cell proliferation and invasion. Uch as PI3K/Akt/mTOR, MAPK/ERK and the like.

In addition to its cancer-promoting effect, LINC02560 was found to have the function of regulating cancer cell metabolism in hepatocellular carcinoma [25]. By means of metabonomics and isotope labeling, It was found that overexpression of LINC02560 could significantly affect the metabolic processes of glycolysis, fatty acid synthesis and oxidative phosphorylation in hepatocellular carcinoma cells。 These changes in metabolic processes not only provide sufficient energy and raw materials for the rapid proliferation of cancer cells, It also promotes immune escape and drug resistance of cancer cells [26]. Further studies have found that LINC02560 regulates genes and transcription factors related to metabolism, such as HIF-1α, PPARγ and so on.To achieve its metabolic regulation.

LINC02560 is also involved in immune escape in hepatocellular carcinoma. Zhu Zhijian used immunohistochemical and flow cytometry techniques. It was found that the high expression of LINC02560 could significantly inhibit the infiltration and activation of immune cells in hepatocellular carcinoma tissues. Thereby weakening the body’s anti-tumor immune response [27]. This finding suggests that LINC02560 regulates the function and activity of immune cells. Thereby helping cancer cells to escape the immune surveillance and elimination of the body. Further studies have found that LINC02560 regulates genes and transcription factors related to immune escape, such as PD-L1, CTLA-4, etc. To achieve its immune escape effect.

Through the technical means of drug sensitivity test and gene knockout, It was found that the high expression of LINC02560 could significantly enhance the resistance of hepatocellular carcinoma cells to chemotherapeutic drugs. And reduce its sensitivity to chemotherapeutic agents [28]. This finding suggests that LINC02560 regulates genes and transcription factors related to drug resistance and chemosensitivity. Such as ABC transporters and apoptosis-related genes, thus affecting the therapeutic effect of hepatocellular carcinoma. Further studies have found that by inhibiting the expression or function of LINC02560,It can significantly improve the sensitivity of hepatocellular carcinoma cells to chemotherapy drugs and reduce their drug resistance [29].

## Regulation mechanism of GLI2 in hepatocellular carcinoma

### Basic composition and function of GLI2

GLI2 is a key component of Hedgehog (Hh), which plays a vital role in a variety of biological processes. Including embryonic development, tissue repair and the occurrence and development of tumors. In HCC, the abnormal regulation of GLI2 is closely related to malignant biological behaviors such as proliferation, invasion, migration and drug resistance of cancer cells [30].

#### Basic components of HH signaling pathway

Hh signaling pathway, as a highly conserved signaling pathway, consists of a number of highly regulated molecular components. This pathway is mainly transcribed by Hh ligand family members, PTCH transmembrane receptors, SMO seven transmembrane proteins, SUFU transcriptional repressor proteins, and GLI zinc finger Factors weave a complex regulatory network together.

The Hh ligand specifically comprises three isomers of IHH (Indian hedgehog protein), DHH (desert hedgehog protein) and SHH (sonic hedgehog protein), They act as signal promoters and trigger a cascade of signal transduction by specifically binding to the PTCH receptor on the cell membrane. PTCH, a receptor protein with a twelve-transmembrane structure, showed a significant inhibitory effect on SMO protein at normal conditions. However, Hh ligand binding reverses this inhibitory effect, causing SMO protein to accumulate on the cell membrane and transition to an activated state.

SMO protein, as a key regulatory node in the Hh pathway, the change of its activation state is crucial to the transduction of downstream signals. Activated SMO can block the interaction between SUFU protein and GLI transcription factors, and then relieve the transcriptional repression of SUFU on GLI.SUFU, a nuclear repressor protein, negatively regulates the transcriptional activity of GLI transcription factors by binding to their C-terminal domains. Activation of SMO disrupts this balance, allowing GLI transcription factors to be released and enter the nucleus.

The GLI transcription factor family includes three members, GLI1, GLI2 and GLI3, which are the final effectors of the Hh pathway. It plays a key role in transcriptional regulation in the nucleus. Once in the nucleus, GLI transcription factors can recognize and bind to specific DNA sequences, thereby regulating the transcriptional activity of downstream target genes. These target genes are widely involved in the regulation of cell proliferation, differentiation, apoptosis, migration and other biological processes. It is of great significance to maintain the homeostasis of the body and respond to environmental changes.

#### Function of GLI2

As a key component of Hh, GLI2 plays a crucial role in a variety of biological processes. During embryonic development, Hh, especially GLI2, precisely regulates the core processes of cell proliferation, differentiation and apoptosis. It is deeply involved in the formation and development of various tissues and organs, and ensures the normal construction and function of organisms. When tissues are damaged, Hh responds rapidly and GLI2 is activated [31] to promote cell proliferation and migration. Accelerate the repair and regeneration of tissues, which is essential for maintaining the homeostasis and recovery of organisms. However, in recent years, many studies have revealed the important role of Hh, especially GLI2, in the occurrence and development of tumors. In HCC, the abnormal regulation of GLI2 has become a key driver of malignant biological behavior of cancer cells. Specifically, GLI2 can upregulate genes closely related to cell proliferation, such as the kinesin gene KIF20A [32]. Thereby accelerating the malignant proliferation of liver cells. Meanwhile, GLI2 also regulates the expression of matrix metalloproteinases (MMPs) and other genes, Significantly enhance the invasion and migration of cancer cells [33]. These findings not only deepen our understanding of GLI2 function, It also provides valuable clues and directions for revealing the morbidity mechanism of hepatocellular carcinoma, exploring new therapeutic targets and improving the therapeutic effect.

### Abnormal activation of GLI2 in hepatocellular carcinoma

The abnormal activation of GLI2 in HCC is a complex and critical process, which involves multiple levels of regulatory mechanisms. Including gene mutation, epigenetic modification, abnormal signal transduction and interaction with others. These abnormal activation mechanisms promote the malignant transformation of hepatocytes and the development of tumors.

#### Gene mutation and abnormal activation

The mutation of GLI2 gene is one of the important reasons for abnormal activation in hepatocellular carcinoma. These mutations occur in the coding region of the GLI2 gene, resulting in changes in the structure or function of the GLI2 protein. Thereby causing abnormal activation under normal physiological conditions. For example, some mutations significantly increase the transcriptional activity of GLI2 protein or make it more sensitive to upstream signals. Thus promoting the proliferation and invasion ability of cancer cells [34]. In addition, mutations in other genes related to GLI2 (such as PTCH, SMO, etc.) Also lead to abnormal activation. It further aggravates the malignant transformation of hepatocytes.

#### Epigenetic modification and abnormal activation

In addition to gene mutations, epigenetic modifications are also important factors contributing to the abnormal activation of GLI2 in hepatocellular carcinoma. Epigenetic modifications include DNA methylation, histone modification and non-coding RNA regulation. They regulate the activity of transcription factors by affecting the transcription and expression levels of the GLI2 gene. In hepatocellular carcinoma, the promoter region of the GLI2 gene is demethylated, resulting in enhanced transcriptional activity of the GLI2 gene. This in turn promotes the abnormal activation of GLI2 [16]. In addition, some non-coding RNAs (such as lncRNA and miRNA) also affect the activity of transcription factors by regulating the expression level of GLI2 gene. These aberrant changes in epigenetic modifications provide a new perspective and explanation for the aberrant activation of GLI2 in HCC.

#### Signal conduction abnormality and abnormal activation

Aberrant activation of GLI2 in hepatocellular carcinoma also involves abnormalities in signaling. These include abnormal activation of upstream signaling molecules, abnormal expression of downstream target genes and imbalance of internal regulatory mechanisms of transcription factors. For example, aberrant secretion of Hh ligand or mutation of the receptor PTCH results in aberrant activation of the SMO transporter, Which in turn promotes the abnormal conduction of GLI2 [35]. In addition, loss of function of SUFU repressor proteins or aberrant expression of GLI transcription factors also results in aberrant activation of transcription factors. These abnormal conduction mechanisms contribute to the malignant transformation of hepatocytes and the development of tumors.

#### Interaction with other transcription factors

The abnormal activation of GLI2 in HCC also interacts with other transcription factors, such as Wnt and TGF-β.These interactions further aggravate the malignant transformation of hepatocytes and the development of tumors by affecting the upstream or downstream regulatory mechanisms of GLI2. For example, the abnormal activation of Wnt regulates the activity of GLI2 by affecting the expression and activity of β-catenin [36]. These findings not only reveal the complex regulatory mechanism of GLI2 in HCC, It also provides a new idea for finding new therapeutic targets and improving the therapeutic effect.

### Regulatory mechanism of GLI2 in hepatocellular carcinoma

The regulation mechanism of GLI2 in HCC is a complex and in-depth research area, which involves multiple levels of interactions and regulatory networks. The upstream regulation, downstream effect and interaction with others of GLI2 will be discussed in the following. To explore the regulatory mechanism of GLI2 in hepatocellular carcinoma in detail.

#### Upstream regulation mechanism of GLI2

In hepatocellular carcinoma, the upstream regulatory mechanism of GLI2 mainly involves the components of Hh ligands, receptors and transporters. Abnormal secretion of Hh ligands such as SHH or mutation of the receptor PTCH results in abnormal activation of the SMO transporter, Thereby promoting the transcriptional activity of GLI2. In addition, loss of function of SUFU inhibitory proteins also results in abnormal activation of GLI2.It is worth noting that recent studies have shown that. Epigenetic modifications such as DNA methylation and histone modifications also play an important role in the upstream regulation of GLI2. For example, demethylation of the GLI2 promoter region leads to an enhancement of its transcriptional activity, which exacerbates the malignant transformation of hepatocytes.

#### Downstream effect mechanism of GLI2

As a key transcription factor of Hh, GLI2 exerts its oncogenic effect by regulating the expression of downstream target genes in hepatocellular carcinoma.GLI2 can up-regulate the expression of genes related to cell proliferation, invasion and migration. Uch as kinesin gene KIF20a and matrix metalloproteinases (MMPs).The abnormal expression of these downstream target genes promotes the malignant proliferation and invasion of hepatocytes. In addition, GLI2 interacts with other transcription factors, such as FoxM1, to regulate the expression of downstream genes. It further aggravates the malignant transformation of hepatocytes.

#### Interaction of GLI2 with others

In hepatocellular carcinoma, there is a complex interaction between GLI2 and others, such as Wnt, TGF-β, and Hippo. These interactions exacerbate the malignant transformation of hepatocytes by affecting the upstream regulation or downstream effects of GLI2.For example, the abnormal activation of Wnt regulates the transcriptional activity of GLI2 by affecting the expression and activity of β-catenin [37]. Similarly, the abnormal activation of TGF-β also affects the expression and activity of GLI2 by affecting the phosphorylation and nuclear translocation of Smad protein. In addition, the deregulation of Hippo also regulates the downstream effects of GLI2 by affecting the expression and activity of transcription factors such as YAP/TAZ [38].

#### Therapeutic targets of GLI2 in hepatocellular carcinoma

In view of the important role of GLI2 in hepatocellular carcinoma, therapeutic strategies targeting GLI2 or its upstream and downstream regulators have become new therapeutic targets. For example, blocking the activation of GLI2 by inhibiting the ligand, receptor, or transporter of Hh, Or inhibit the malignant proliferation and invasion ability of hepatocytes by inhibiting the downstream target genes of GLI2.In addition, treatment strategies targeting GLI2 and other interactions also have potential clinical applications.

## The mechanism of LINC02560 regulating GLI2

### Interaction of LINC02560 with GLI2

LINC02560, an lncRNA with aberrant expression in a variety of cancers, exhibits a significant up-regulated expression pattern in HCC, It suggests that it plays an important role in the occurrence and development of hepatocellular carcinoma. In particular, the interaction between LINC02560 and GLI2 provides a new perspective for understanding the morbidity mechanism of hepatocellular carcinoma.LINC02560 has been shown to regulate GLI2 through a variety of mechanisms. First, LINC02560 can directly bind to GLI2 protein to form a complex. This binding regulates the expression of its downstream target genes by affecting the transcriptional activity or stability of GLI2, And then affect the malignant biological behavior of hepatocytes. Secondly, LINC02560 also regulates the expression level of GLI2 gene by affecting the epigenetic modification of its upstream regulatory elements. Such as recruiting specific epigenetic modification enzymes to the promoter region of the GLI2 gene, changing its methylation or histone modification status [39]. In addition, LINC02560 not only directly interacts with GLI2, but also interacts with others (such as Wnt, TGF-β, etc.).Can jointly regulate and control the malignant biological behaviors of liver cells, This interaction occurs through upstream regulators or downstream effectors that affect GLI2 [40]. Finally, LINC02560 also regulates the location and activity of GLI2 in the nucleus by binding to GLI2 and affecting its nucleoplasmic shuttle process. It further affects the transcriptional activity of downstream target genes. In conclusion, LINC02560 regulates GLI2 through complex mechanisms and plays an important role in the occurrence and development of hepatocellular carcinoma. It provides an important clue for finding new therapeutic targets and improving the therapeutic effect.

### Molecular mechanism of GLI2 regulation by LINC02560

LINC02560 is an lncRNA that is significantly up-regulated in HCC. Its interaction with GLI2 provides a new perspective for understanding the morbidity mechanism of hepatocellular carcinoma. When exploring the mechanism of GLI2 regulation by LINC02560, we found that its molecular mechanism involves complex regulation at multiple levels.

LINC02560 forms a functional complex by directly binding to the GLI2 protein [41]. This binding not only stabilizes the structure of the GLI2 protein, but also enhances or inhibits the transcriptional activity of GLI2 by changing its conformation. Specifically, LINC02560 acts as a co-activator of GLI2, promoting its ability to bind to DNA. Thereby upregulating the transcription level of the downstream target gene. These target genes are usually closely related to malignant biological behaviors such as cell proliferation, invasion and migration. Therefore, the binding of LINC02560 to GLI2 directly promotes the malignant transformation of hepatocytes.

In addition to direct binding, LINC02560 regulates the expression level of the GLI2 gene by affecting its epigenetic modification. Epigenetic modifications include DNA methylation, histone modification and so on. They regulate gene expression by altering gene accessibility or transcriptional activity.LINC02560 recruits specific epigenetic modification enzymes to the promoter region of the GLI2 gene, It regulates the transcriptional activity of GLI2 by affecting its methylation status or histone modification pattern.This regulatory mechanism leads to the abnormal increase of GLI2 expression in hepatocellular carcinoma, which aggravates the malignant biological behavior of hepatocytes.

In addition, LINC02560 regulates GLI2 through other interactions. In hepatocellular carcinoma, there is a complex interaction network among multiple genes, which jointly regulate cell growth, differentiation and apoptosis.LINC02560 is a key node in these interactive networks. It affects the upstream regulators or downstream effectors of GLI2 through interaction with other factors such as Wnt, TGF-β, etc [42]. This interaction makes the regulation of GLI2 in hepatocellular carcinoma more complex and diverse.

### Biological significance of GLI2 regulated by LINC02560 in hepatocellular carcinoma

LINC02560, an lncRNA with abnormal expression in HCC, The mechanism of GLI2 regulation has a profound biological significance in the occurrence and development of hepatocellular carcinoma. As a key transcription factor of Hedgehog, GLI2 plays an important role in many cancers. The interaction between LINC02560 and GLI2 further enriches our understanding of the mechanism of morbidity in hepatocellular carcinoma.

In hepatocellular carcinoma, LINC02560 regulates GLI2 through a complex mechanism. And then affect the biological processes of cell proliferation, invasion, migration and apoptosis. Specifically, LINC02560, as a regulator of GLI2, regulates the expression of downstream target genes by affecting their transcriptional activity or stability. These target genes are often closely associated with key biological processes such as cell cycle regulation [43], extracellular matrix remodeling [44], and angiogenesis However, their abnormal expression directly promotes the malignant transformation of hepatocytes and tumor progression.

The biological significance of GLI2 regulated by LINC02560 in hepatocellular carcinoma is also reflected in its impact on the tumor microenvironment. The tumor microenvironment is a complex ecosystem comprising tumor cells, immune cells, blood vessels, lymphatic vessels, and other cellular components [45].They regulate tumor growth and metastasis through complex interactions.LINC02560 can affect the immune cell infiltration, angiogenesis and inflammatory response in the tumor microenvironment by regulating GLI2 [46]. Thus providing a favorable microenvironment for tumor growth and metastasis [47].

In addition, LINC02560 regulation of GLI2 also affects the therapeutic effect and prognosis of hepatocellular carcinoma [48]. Because GLI2 plays an important role in various cancers, targeted therapies targeting this pathway have become a hot topic of current research. As a regulator of GLI2, the expression level of LINC02560 is closely related to the therapeutic effect and prognosis of hepatocellular carcinoma [49]. By detecting LINC02560 expression levels, we can predict the response and prognosis of patients to specific therapies [50]. So as to provide an important basis for the formulation of individualized treatment [51].

## Conclusion

LINC02560 promotes the proliferation, invasion, and immune escape of hepatocellular carcinoma (HCC) by directly binding to GLI2 protein, regulating its epigenetic modifications, and interacting with other signaling pathways. Its high expression is significantly correlated with poor patient prognosis and chemotherapy resistance, suggesting that the LINC02560/GLI2 axis may be a potential target for precision treatment of HCC. This study not only deepens the understanding of the pathogenesis of HCC, but also provides new directions for the development of targeted therapy strategies.

## Data Availability

No datasets were generated or analysed during the current study.
